# Experiences of Greek women of water immersion during normal labour and birth. A qualitative study

**DOI:** 10.18332/ejm/92917

**Published:** 2018-07-12

**Authors:** Angeliki Antonakou, Erifyli Kostoglou, Dimitrios Papoutsis

**Affiliations:** 1 Midwifery Department, Alexander Technological Educational Institute of Thessaloniki, Thessaloniki, Greece; 2 Department of Obstetrics and Gynaecology, Shrewsbury and Telford Hospital NHS Trust, Telford, United Kingdom

**Keywords:** childbirth, water birth, water immersion, alternative birthing methods

## Abstract

**INTRODUCTION:**

There is scarce information on water births in Greece, as few women labour and give birth in water. The Greek public health system does not provide water immersion as a birthing option, and so women can only experience this option in private healthcare settings. The aim of this study was to explore the key concepts and themes identified from an analysis of the experiences of women who laboured and gave birth immersed in water.

**METHODS:**

This was a qualitative study involving twelve women who used water immersion during labour, of which nine had also a water birth. Individual interviews were conducted and their content was analysed using thematic analysis.

**RESULTS:**

Three main themes were identified: *Water use as a natural way of birth, Mixed messages from the healthcare professionals, and Partner’s supportive role during water birth*. All women reported a positive birth experience and water immersion helped them cope with the pain of labour. They felt greatly empowered following birth and this contributed to successful breastfeeding for more than one year, in the majority of cases. They reported difficulties in finding healthcare professionals that were in support of their choices. The women felt highly supported by the partner’s role.

**CONCLUSIONS:**

Labouring and giving birth immersed in water was met with great satisfaction by all women. The findings of this study have added to the current body of midwifery knowledge on how water immersion can improve a woman’s experience and so support a normal birth outcome.

## INTRODUCTION

Following the publication in 1983 by Michel Odent who reported on the water birth outcomes of one hundred women, labouring and giving birth in the water became popular in Europe^1^. That study advocated that there was no risk attached to the immersion in water and that the use of drugs and the rate of intervention in parturition were both reduced. A decade later, the Changing Childbirth report issued from the Department of Health in 1993 recommended that all maternity units in the United Kingdom should provide women with access to a birthing pool facility^2^. In 1994, the Royal College of Midwives published a statement that emphasized that the role of the midwife should include the ability to support and facilitate water immersion in labouring women^3^. Today, water immersion in labour and birth has been fully integrated in the policy of maternity services provided in the United Kingdom and as a standard birthing option in Midwifery practice^4^.

In Greece over the past few decades, birth has become highly medicalised. This means that birth takes place within hospital environment settings in either secondary or tertiary general hospitals of the Greek State-funded National Health Service (NHS) or in tertiary private hospitals^5^. Unfortunately, there are no primary healthcare settings, no community midwives or midwifery-led birthing units in the current Greek (public or private) healthcare system, and home births represent less than 0.9% of total births^5^. Maternity care in the Greek NHS is free to all women and it covers all costs for antenatal care, childbirth and postnatal care. Maternity care in private hospitals is where a woman has an identified consultant obstetrician and midwife, who are paid a fee usually through a private insurance system that also covers any other maternity-care related costs. Antenatal, intrapartum and postnatal care to women is provided by a combined team of doctors and midwives and giving birth in a Greek hospital is performed in the presence of both at childbirth^6^. Labour is medicalised with high rates of interventions, such as the use of epidural analgesia, continuous fetal monitoring and caesarean section rates^7^ reaching 54.8%. Birthing units in the Greek NHS do not offer water immersion during labour or birth. On the other hand, the first birthing pool at a private maternity hospital was installed back in 1999. Nowadays, almost all birthing units in the private healthcare system offer birthing pool facilities. This fact alone, although there are no official statistics about the potential demand for women in Greece to labour and birth in the water, shows the increasing demand for this kind of facilities.

There are qualitative reports in the literature that explore women’s subjective experiences of labouring and giving birth in the water. It has been shown that women who chose to use water immersion during their labour reported increased levels of satisfaction and greater sense of control^8-11^. Women reported feeling more confident after a water birth^10,12,13^ , with a greater emotional well-being postnatally^14^. While most reports concerning the use of water immersion in labour and birth originate from western countries, there are limited reports from Mediterranean countries where maternity services are offered in greatly medicalised environments^15,16^.

The evidence on the benefits and potential risks of water immersion during labour and birth has been mostly generated by observational studies. A Cochrane Database systematic review published in 2018 analysed fifteen randomised controlled trials from different countries with the objective of assessing the available evidence about water immersion during labour and birth^17^. The review concluded that labouring in water may reduce the number of women having an epidural. There was no evidence that labouring in water increases the risk of an adverse outcome for women or their newborns. Also, it did not appear to affect the mode of birth or the number of women having a serious perineal tear^17^. Nevertheless, in all these studies the study design did not make it possible to capture the experiences of women with regards to water immersion during labour and birth.

Since observational studies cannot capture women’s experiences with water immersion and there are no data on this subject for Greece, the primary endpoint of this study was to explore from a qualitative perspective the experiences of Greek women who used water during labour and birth. To the best of our knowledge, this is the first qualitative study reporting on the experience of women labouring and giving birth in water in Greece.

## METHODS

This is a qualitative study with the use of thematic analysis described by Braun and Clarke as its main research tool^18^. Thematic analysis has been reported in the literature and provides a robust and systematic framework for the coding of qualitative data to determine patterns and themes/sub-themes across the data set in relation to the research questions posed by the researchers^19^.

For the purposes of this study, we also conducted a literature review by searching the MEDLINE and EBSCO databases for the years 2000-2017 with the use of the following text words: *water birth, water immersion, childbirth, alternative birth*. The references of the relevant articles were also searched to capture any other reports that were not readily identified in the electronic search.

The study received ethical approval from the scientific board of the ‘Alexander’ Technological Educational Institute of Thessaloniki in Greece.

### Data collection

Women were publicly invited to participate in this study through an open invitation that was posted on the website of the Hellenic Psychoprophylaxis Society (http://birthscientist.gr/), which is a scientific society with the primary aim to promote normality during childbirth in Greece. The only inclusion criteria were the mothers’ willingness to share their experience on using water immersion during their labour and birth, and their ability to speak and clearly understand the Greek language. Women who responded to this invitation were informed about the study protocol, its aims and the voluntary character of their participation. Once an informed consent was given, a code name (pseudonym) was assigned to each participant to ensure their anonymity.

Individual face-to-face interviews were arranged and were conducted during the period January to July 2014. The individual interviews ranged from thirty minutes to a maximum of one hour. At the interviews there was a series of openended questions that were developed by the researchers to be used as a guide (Table 1). They were designed to keep the interview sessions on track while exploring at the same time the thematic issues relevant to the research questions. These open-ended questions were intended to provide a structure to stimulate discussion and not rigidly dictate the line of the interview. The interview guide was initially tested with two participants to determine whether the questions were clearly understood and interpreted and to establish face validity. The pilot testing was considered successful and no changes were therefore made to the set of questions.

**Table 1:** Open-ended questions for interviews

*Question*
What was the source of the information you had about the use of water during labour and at birth?
Why did you choose to use water during labour and at birth?
What was your birthing experience like?
Do you feel you were well supported as to your choice of birthing?
Any other issues you feel you would like to highlight?

Recruitment of women was stopped when thematic saturation was considered by the researchers to have been broadly achieved, and therefore any further interviews were not expected to yield additional important information for this study. At the time point of discontinuation of recruitment, there were eighteen women who had responded to the invitation to participate in the study, of which twelve were interviewed as the other six lived far from Athens where the research and interviews took place.

### Data analysis

For the purposes of the study, we used the six-step approach for a thematic analysis as described by Braun and Clarke^18^. In the initial phase of the thematic analysis, the researchers organised and verbatim transcribed the interviews they had with the women, along with the initial thoughts and reflections they had immediately after the interview. In the next phase, the researchers conducted ‘repeated reading’ of all the available material, which results in data immersion allowing the researcher to achieve familiarity with the data. Next, codes were assigned to interesting features after repeated reading of the transcribed interviews. When data were considered relevant to codes they were all sorted under the same code. Following this phase, all codes were incorporated into potential themes. Themes were subsequently checked for coherent, consistent and clearly identifiable distinctions, and given short names (or phrases) that conveyed an immediate indication of their contents.

**Figure 1:**
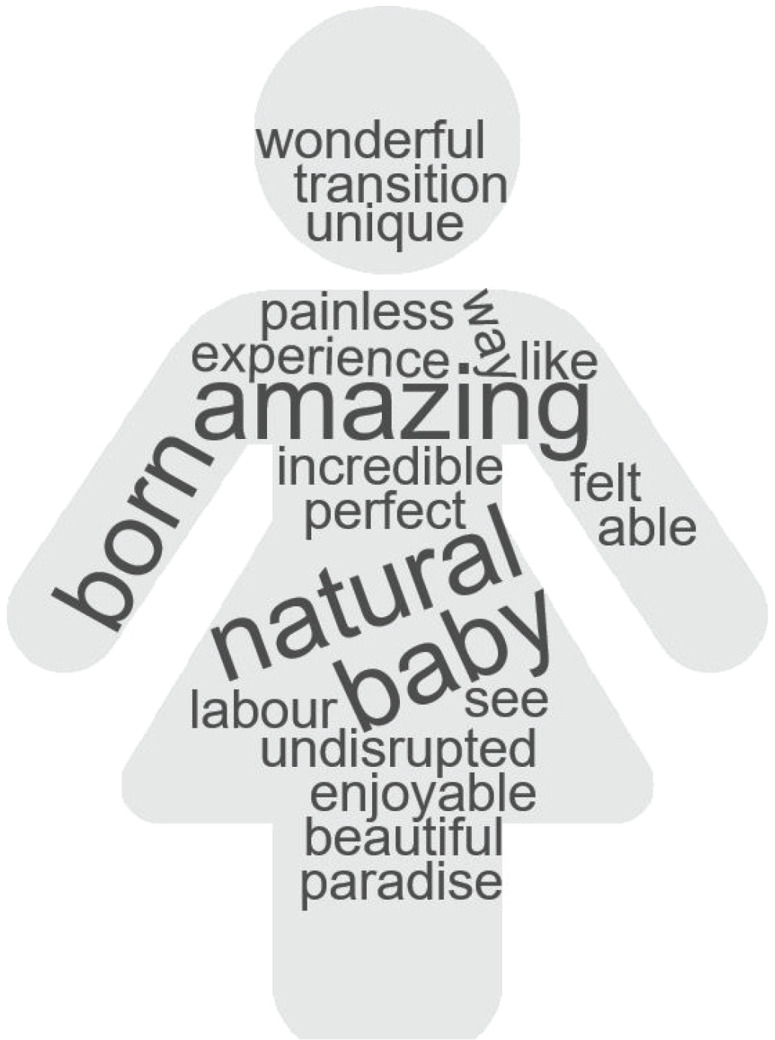
The verbal responses of women about their water immersion experience during labour and at birth. The larger the font size in the word cloud, the more frequent it has been used and re-iterated by women

In the last phase of the analysis, we chose to present extracts from the interview transcripts to illustrate elements of the themes and present examples of the points being made. We also chose to provide a visualization of the women’s responses in a word-cloud figure with the use of a software tool (https://www.wordclouds.com/). In Figure 1, the larger the font size in the word cloud, the more frequent it was used as a response and re-iterated by the women.

In order to reduce bias, increase credibility and reconcile any inconsistencies, the researchers had an ongoing critical discussion on the findings and themes.

### Participants

The mean age of the 12 women in our study was 38 years (SD: 4.63, range: 29–45), with 6 being primiparous, 5 giving birth for the second time and one for the third time. All women were Caucasian and married to male partners. Seven women were university graduates of which 4 had post-graduate degrees (MSc or PhD). Ten out of twelve women used water immersion during their labour for the very first time, while two had used this method before. Nine out of twelve women gave birth in the birthing pools, whilst three women used water immersion only for the first stage of labour. When interviewed all women were at least one year postpartum (range: 1–3 years).

### Identified themes and sub-themes

The main themes have been labeled as: *1) Water use as a natural way of birth, 2) Mixed messages from the healthcare professionals, and 3) Partner’s supportive role during water birth*. The main themes and sub-themes that were identified through the thematic analysis are presented in Table 2.

**Table 2:** Main themes and sub-themes

*Main themes*	*Sub-themes*
Water use as a natural way of birth	Excellent birth experience No significant perineal tears Breastfeeding
Mixed messages from healthcare professionals	-
Partner’s supportive role during water birth	-

### Water use as a natural way of birth

#### Excellent birth experience

The great majority of women reported that they were totally satisfied with the experience and went on to describe it with enthusiastic words and phrases, like ‘amazing experience’, ‘perfect’, ‘incredible’, ‘wouldn’t change a thing’. They also used words like ‘beautiful’, ‘unique’ or ‘wonderful’ to describe their experience. They felt that *they* were in control of their body and that they played an active role during their labour. They also were happy they could easily change positions in the birthing pool and felt more relaxed due to the warm water. According to S.: ‘*Being in the water was a perfect experience. It was what I needed to help me deal with the pain. The most emotional moment was when the amniotic fluid joined the water in the birthing pool*’.

*‘When I got in the water I felt like being in paradise!! It was amazing!!’, commented St., while A. said: ‘… I was able to see my baby being born, that is irreplaceable for me’*.

Interestingly enough, those women who did not deliver in the water expressed similar feelings of satisfaction about the overall experience when compared to the women who had a water birth.

K. commented: ‘*I am very satisfied with my experience although I did not manage to deliver in the water. I felt safe, I was calm and I did not feel any pain… . I felt relief in the water*’.

All women were keen on recommending water immersion during labour and birth to friends and relatives. They commented that water immersion is a ‘natural way for the baby to be born’ and the perfect way to an ‘enjoyable, undisrupted painless labour’. They also commented that it makes childbirth a ‘nice, calm, and relaxed experience’.

All women stated that they chose a water birth because they wanted to have a natural birth without interventions and no use of pharmaceutical analgesia.

*According to I.: ‘the use of water during childbirth is usually a positive, nice and painless experience without any use of medication’*.

*J. stated: ‘We had talked about it with my midwife during prenatal classes… . I felt this was what I had to do in order to give birth naturally’*.

Women with a previous experience of water immersion also commented on the positive fact of not having to be connected to a monitor and the ability to easily change positions in the birthing pool. While other women also mentioned that water offers the baby a ‘natural transition from the uterus to the mother’s arms’, and this enhanced their positive experience.

All these positive responses of women with regards to their water immersion experience are illustrated in the word cloud of Figure 1.

#### No significant perineal tears

All women, without being specifically asked at the interview and in response to the question of whether they wanted to highlight any other issue, talked about not having an episiotomy and having only superficial first-degree perineal tears or no tears at all. They were all really happy and proud with this achievement.

A. commented: ‘I was amazed to hear that I didn’t have any tears… all my friends had an episiotomy when they delivered! I am sure being in the water, made the difference!’.

#### Breastfeeding

During the interviews, all women reported that they started breastfeeding immediately after giving birth. After such a natural experience they wanted to hold and feed their baby. The neonate was placed immediately in their arms and within the first thirty to sixty minutes started sucking. The midwives’ guidance was highlighted, as they supported the initial contact of mother and baby by encouraging early skin-to-skin contact and breastfeeding. Even more interesting, all women who were interviewed were reportedly breastfeeding their babies for a year or more following birth, and some were still breastfeeding by the time of the interview. All women reported that they had complete trust in their midwife during labour and birth in the water.

*St. mentioned: ‘My midwife was so supportive, I felt I could ask her anything and anytime, and that helped me a lot with the breastfeeding process… giving birth in the water helped develop a deep relationship of trust with my midwife…’*.

*S. said: ‘I started breastfeeding in the delivery room… . It felt so natural… .We are 16 months old and still breastfeeding! I know I couldn’t have made it so far, if I didn’t have the support from my midwife! Especially when I felt I didn’t have enough milk, she was always there to help me, guide me and reassure me like she did when I was in the water labouring!!!’*.

*Cl. mentioned: ‘I wanted to breastfeed my baby but my friends were saying it would be difficult, with the stitches and everything…. I was feeling fine after giving birth… she latched on immediately and hasn’t stopped… . She is now 13 months old.’*.

### Mixed messages from the healthcare professionals

All women reported that they were familiar with the concept of water immersion during labour and birth from the early stages of pregnancy. Their source of information was mostly from the internet but also from discussions during the prenatal classes with others who had a similar previous experience, or with friends and relatives who had tried the method in the past. Nevertheless, they stated that they would use water immersion during labour and birth only after being informed by their midwife or their obstetrician, and after discussing it with their partner.

Five out of the twelve women mentioned that they struggled to find healthcare professionals willing to facilitate water immersion during labour and at birth. Moreover, during their contacts with different healthcare professionals they received conflicting information about the safety and the benefits of this method, but they themselves were committed to try it. Amongst these healthcare professionals, all midwives who were approached were reportedly supportive of water immersion during labour and birth. In contrast, the majority of obstetricians whose opinion was sought by the women of this study had no knowledge or were negatively predisposed to water births.

M. stated: ‘I was told that it doesn’t make any difference (… being in the water) but I saw the videos on the internet and spoke to a couple who had tried it, so I was determined at least to have the chance to use it during the birth of my son’. Cl. said: ‘The first doctor that I went to when I found out I was pregnant, dismissed my wishes to have a water birth… he said that it could be dangerous for me and my child. I sought the advice of another doctor and was reassured that no such danger existed provided my pregnancy would remain uncomplicated… in the end, I did it and gave birth in the water!!!’.

### Partner’s supportive role during water birth

The partner’s role during childbirth was brought up in all interviews. All women commented on how important it was for them to have the ‘positive support’, ‘encouragement’ and ‘reassurance’ from their birthing partner. In all cases, the partner was present at birth, had an active role and in some cases even cut the umbilical cord. His role during the decision making was also commented upon. In some cases, after being informed by the midwife or the obstetrician, he was the one to help his pregnant partner to overcome any hesitation and supported her throughout the process.

*‘My partner was very happy and enthusiastic about the idea of water birth’, said E*.

*O. commented: ‘I was giving birth for the first time, so I was a little worried. My husband was able to calm me down. He was very supportive. He helped me move around the pool and breathed with me through the contractions…’*.

*‘I thought I could not do it without an epidural analgesia. My husband helped me a lot…’, said K. and similarly El. commented: ‘My partner’s role was very determinant. He massaged my back in the pool! He helped me so much! I could not have done it without his support!’*.

## DISCUSSION

This was a qualitative study on Greek women who laboured and gave birth in water. Thematic analysis was the main research tool used to capture the key concepts related to their water birth experiences. The first main theme, **Water use as a natural way of birth,** highlights the fact that all women considered water immersion in labour and birth as the most natural way they could give birth. They described an *Excellent birth experience*, noted in our study as a sub-theme. They stated positive feelings about their water immersion experience and commented being in control and having an active role while remaining calm and relaxed during labour. The available literature, on a global scale, suggests that water immersion increases maternal satisfaction levels and the sense of control, leading to greater emotional stability postpartum^10-12,14^. Moreover, labour in water has been shown to reduce stress hormones and promote the release of endorphins, therefore allowing for better labour progress and greater maternal calm and relaxation^20^.

In this study it is interesting to note that even the women who did not give birth in the water had high levels of satisfaction and were eager to pass on their experience to other women to help them achieve a natural, enjoyable and undisrupted labour. This finding shows that when women feel empowered, even though not having the type of birth they planned, they still feel satisfied and contented with their experience. This finding is in line with the Royal College of Midwives initiative in 2011, which aimed to promote water births as a means of empowering women and normalising birth^21^. There are reports in the literature that empowerment is a characteristic that may influence a woman’s experience at birth^22^. The concept of empowerment comprises multiple constructs such as: the ability of the woman to access and utilize healthcare resources, the woman’s mobility and autonomy, and the ability of exercising an informed choice among a series of alternatives^22,23^. Water immersion for labour and birth was a means of empowerment for the women of our study, as this supported their autonomy and gave them the chance to exercise choice against the alternative of a medicalised hospital birth.

The women in our study reported that they sought to use water for labour and birth as a natural way of giving birth. Giving birth in a Greek hospital follows the example of other countries where the setting is highly medicalised^24^. In Greece, women are subjected to routine intravenous infusions and oxytocin in labour. Even without obstetric complications they are encouraged to have continuous electronic fetal monitoring and epidural analgesia. Moreover, labour will most often be in the dorsal position and birth in lithotomy. There are reports that women in highly medicalised birthing environments found the experience traumatising and were provided with no choice when choice was important to them during their labour^25^. In this context, the women in our study chose water use in labour and birth as an alternative option of care that more appropriately met their needs.

The women in our study reported that they were able to cope with the pain of labour and that water offered relief to them. There are reports that the ease of mobility in the birthing pool and the buoyancy of water allows a better flexed fetal head position in the pelvis, a shorter labour and less painful contractions^26-30^. Other studies report that water immersion in labour may facilitate the neuro-hormonal interactions of labour, thus reducing pain levels^31,32^. In addition, labouring in water enhances the physiology of childbirth and induces the release of the endogenous endorphins, which act as the natural opiates of the body producing a pain-relief effect^1,33^.

The sub-theme of No significant perineal tears, which falls under the main theme of **Water use as a natural way of birth,** described the fact that women in our study commented positively that they experienced minimal perineal trauma during water birth. Of course, this does not imply that women who have a perineal tear or an episiotomy are not experiencing a normal birth. Nevertheless, this element was so significant to the women of our study that they all mentioned it unsolicited. Mainly they wanted to comment on the fact that other women, who they knew and did not have a water birth, had perineal trauma at their childbirth. It has been well documented that warm water increases the elasticity of the perineum resulting in decreased frequency and severity of perineal trauma^17^. Moreover, a recent observational study in Italy, where caesarean section rates and episiotomy rates are also high, showed a significant decrease in perineal trauma rates for women laboring in the water^16^.

The sub-theme of *Breastfeeding* highlighted the fact that water birth as a way of normal birth allowed women to start breastfeeding immediately within the first hour after giving birth. Moreover, all women breastfed exclusively for the first six months postpartum and most of them continued for more than a year. It has been reported that the facilitation of women to labour and birth in the water offers midwives the opportunity to form a therapeutic rapport with women and the chance to empower them in realizing their potential^4,34^. In our study, all participants highly commended their midwives and acknowledged the therapeutic relationship that was formed between them during the water-use experience. This finding along with the breastfeeding support and guidance offered by the midwives^35^ may explain the prolonged breastfeeding time periods identified in our study.

The second main theme, **Mixed messages from the healthcare professionals,** represents the dissatisfaction that women felt with the difficulty in accessing information about water birth, and the struggle they had in identifying healthcare professionals that were willing to support their choices. It has been reported that the Internet is an important source of information, with mothers spending approximately three hours a day^36^, and a place for sharing experiences^37,38^. Studies have demonstrated that the Internet is an important source of information on: prenatal dietary advice, physical activity, weight control suggestions during pregnancy, and intrapartum pain management^39,40^. In our study, women obtained much of their information on water birthing from the Internet but nevertheless required the final recommendations from their healthcare professionals.

The midwives in our study were highly commended by all women, however the obstetricians were those who reportedly dismissed the water birthing choices of women and intimidated them by questioning the safety of their choice at birth. This finding has also been reported in other highly medicalised environments where a paternalistic attitude towards the mother exists. In this kind of environment, either obstetricians seem not to have sufficient knowledge and skills to support this birthing option or the model of care provision in the maternity unit is not woman-centered^41,42^. Another reason obstetricians do not support the option of labouring and giving birth in water might be that observational studies in the literature report only the benefits and risks of water immersion but do not capture the experiences of women^17^ to better inform the medical practitioners. It has also been reported that due to little high-quality research on the safety and practicality of water immersion, policies and guidelines informing the practice may lack the evidence necessary to ensure practitioner confidence with this option, thereby limiting both accessibility and women’s autonomy^43,44^.

The third main theme, **Partner’s supportive role during water birth,** explores the role of the partner during labour and birth in the water. The women stated that their birthing partner helped them stay calm and positive throughout the whole experience while offering them a sense of security and familiarity. In our study, the partner was described as being enthusiastic about the idea of water birth. He was actively involved in the labour process in such ways as helping the mother change positions in the pool, massaging her back and assisting her with breathing techniques during contractions. The finding that water immersion during labour gave the partner the opportunity for an active involvement that led to a positive birthing experience, has been reported in a national Maternity survey conducted in England in 2010. This survey showed that the partner’s engagement during childbirth influenced significantly the woman’s uptake of services, her perceptions of care and the maternal outcomes^45^.

### Limitations and strengths

In this qualitative study conducted with only twelve women, the mean age of the women was 38 years, with the great majority being highly educated and all having received private care. It has been reported that older women, with a higher educational and economical status might have higher expectations from the maternity care they receive^46^. Therefore, the views expressed and experiences recounted by the participants may not have been shared by other women. Moreover, birthing pools currently only exist in private hospitals in Greece as the national health system does not offer this birthing option. The women in our study were therefore socio-economically advantaged and the results cannot be extrapolated to the entire Greek population. Also, the interviews were conducted more than a year from the water birth experience and so a recall bias cannot be excluded or accounted for in the analysis. Moreover women were invited via the Internet from a website, therefore those who did not have access to a computer but potentially would have been interested to participate did not have the chance to be included in this study.

The main strength of this study is that its findings are supported by the current literature and so they contribute to current midwifery knowledge. Based on our literature review, and to the best of our knowledge, this is the first qualitative study reporting on the experience of women labouring and giving birth in water in Greece. Also, we included women who didn’t achieve a water birth but had water immersion during labour.

### Areas for future research

Our study highlights the lack of support from obstetricians for the water immersion birthing option in Greece. This lack of support could be attributed to the lack of knowledge and skills on labouring and giving birth in water, to paternalism that has been reported to prevail in hospital environments^41,42^, and to the lack of high quality evidence to inform clinical practice on water births^43,44^. It has been reported that water birth workshops have the potential to significantly enhance the adoption of the midwifery model of care that pursues normality even within a medically dominated hospital birthing environment^47^, such as that in Greek maternity hospitals. In that study^47^, the maternity caregivers were midwives, and therefore the findings cannot be extrapolated to other health professionals such as obstetricians. However, this is an area of potential future research that if corroborated could provide a simple tool of reversing the negative attitude that obstetricians have on water births.

## CONCLUSIONS

This qualitative study, despite inherent limitations, provides insights on the experiences of Greek women with labouring and giving birth in water. The participants of our study were a homogenous group of older, welleducated and able to afford private maternity care women. Therefore, their experiences may not have been shared by other women, especially in Greece where they do not have access to water immersion for labour and birth in public hospitals. Nevertheless, our study highlighted their excellent birth experience, the ability to cope with pain in labour and birth with no significant perineal trauma, and how greatly empowered the majority were following their water immersion experience so that they were able to breastfeed for more than a year. Finally, our study demonstrated the active and supportive role of the partner during labour.

An area for future research that arose from our findings involves the need to engage, inform and educate obstetricians about the safety and benefits of this birthing option, even though they are not the main practitioners of water immersion but nevertheless women do seek their opinion.
